# Sex differences in survival after out-of-hospital cardiac arrest: a meta-analysis

**DOI:** 10.1186/s13054-020-03331-5

**Published:** 2020-10-19

**Authors:** Hao Lei, Jiahui Hu, Leiling Liu, Danyan Xu

**Affiliations:** Department of Cardiovascular Medicine, The Second Xiangya Hospital, Central South University, 139 Middle Renmin Road, Changsha, 410011 Hunan China

**Keywords:** Out-of-hospital cardiac arrest, Sex, Survival, Prognosis, Post-resuscitation care

## Abstract

**Background:**

Out-of-hospital cardiac arrest (OHCA) is a leading cause of sudden cardiac death worldwide. Researchers have found significant pathophysiological differences between females and males and clinically significant sex differences related to medical services. However, conflicting results exist and there is no uniform agreement regarding sex differences in survival and prognosis after OHCA. Therefore, we investigated the relationship between the prognosis of OHCA and sex factors.

**Methods:**

We comprehensively searched the PubMed, Embase, and Cochrane databases and obtained a total of 1042 articles, from which 33 studies were selected for inclusion. The pooled odds ratios (ORs) and 95% confidence intervals (CIs) were estimated using a random-effects model.

**Results:**

The meta-analysis included 1,268,664 patients. Compared with males, females were older (69.7 years vs. 65.4 years, *p* < 0.05) and more frequently suffered OHCA without witnesses (58.39% vs 62.70%, *p* < 0.05). Females were less likely to receive in-hospital interventions than males. There was no significant difference between females and males in the survival from OHCA to hospital admission (OR 0.99, 95% CI 0.89–1.1). However, females had lower chances for survival from hospital admission to discharge (OR 0.59, 95% CI 0.48–0.73), overall survival to hospital discharge (OR 0.73, 95% CI 0.62–0.86), and favorable neurological outcomes (OR 0.62, 95% CI 0.47–0.83) compared with males.

**Conclusions:**

Our results indicate that the overall discharge survival rate of females is lower than that of males, and females face a poor prognosis of the nervous system. This is likely related to the pathophysiological characteristics of females, more conservative treatment measures compared with males, and different post-resuscitation care. However, these findings should be interpreted with caution due to the presence of several confounding factors.

## Background

Out-of-hospital cardiac arrest (OHCA) refers to the sudden loss of heart function outside of the hospital, which leads to complete cessation of systemic circulation. Cardiogenic factors, such as coronary heart disease and cardiomyopathy, are the main cause of OHCA. At present, OHCA is a leading cause of sudden cardiac death worldwide. It is reported that for every 100,000 people in Europe, 1753 people have experienced OHCA that was subsequently treated and registered by the emergency medical service (EMS) [1]. In the USA, approximately 55 out of every 100,000 people have OHCA [2]. Moreover, a considerable number of patients suffering from OHCA do not receive timely treatment from EMS, so they are not recorded. The mortality rate of OHCA remains high. Globally, the discharge survival rate of OHCA is low: approximately 3.0% in Asia, 6.8% in North America, 7.9% in the UK, 7.6% in Europe, and 9.7% in Australia [3]. It is estimated that 275,000 people receive EMS treatment each year in Europe, and only 29,000 people are discharged alive [4]. Previous studies have shown that the prognosis of OHCA is related to factors such as age, primary disease, bystander cardiopulmonary resuscitation, and initial heart rhythm.

Recently, an increasing number of studies have focused on the differences in the prognosis of various diseases between males and females. Studies have shown that there are significant differences in pathophysiology between men and women, and there may be different clinical symptoms and prognoses [5, 6]. Compounding this, research has identified differences in the medical services received by males and females after admission [7, 8]. The medical services include post-resuscitation care and nursing measures. Cardiovascular disease is the most well-studied topic related to studies on sex differences. Various studies have shown that there is a significant correlation between sex and the pathophysiology and prognosis of cardiovascular disease. For example, for coronary heart disease, females are more likely to have non-obstructive microvascular disease and endothelial dysfunction, while males are mainly characterized by the formation of obstructive macrovascular plaques [9]. Females, especially young females, also fare worse than men after acute myocardial infarction [10].

However, it is not clear whether sex differences in heart-related diseases affect the prognosis of OHCA. Multiple studies have addressed possible sex differences associated with OHCA prognosis, but the findings are inconsistent: some studies report no difference between males and females in survival after OHCA [7, 11]; others have reported better survival for males or better survival for females [12, 13]. A meta-analysis published in 2015 showed that the discharge survival rate of female OHCA patients was 10% higher than that of male, but the results of the latest large clinical studies show conflicting trends [14].

Therefore, to address these issues, we conducted a systematic review and meta-analysis to evaluate the relationship between prognosis and sex after OHCA from a multidimensional perspective. Prognostic factors are divided into three categories: baseline characteristics, pre-hospital care, and hospital care. Our findings provide guidance related to narrowing the difference in prognosis between males and females after OHCA.

## Materials and methods

### Study design and database search

The study was designed following the PECOS principle, where P (participant): OHCA patients; E (exposure): female; C (comparison): male; O (outcome): the survival from OHCA to hospital admission, the survival from hospital admission to discharge, overall survival after hospital discharge, and favorable neurological outcomes. We did not limit the search to the type of research or language of the publication.

A comprehensive search was conducted in PubMed, Embase, and Cochrane databases for related publications between 1980 and October 2019. We searched studies on the relationship between sex and prognosis after OHCA. All published studies prior to October 2019 were considered. Details of the search terms are provided in Additional file 1.

### Inclusion and exclusion criteria

All available retrospective cohort studies that compared females and males in the prognosis after OHCA and that had at least one of the following outcomes mentioned in this paper were included. The primary outcome was the survival from OHCA to discharge; the secondary outcomes were the survival from OHCA to hospital admission, the survival from admission to discharge, and favorable neurological outcome. In-hospital cardiac arrest-related research, animal research, basic research, letters, meta-analyses, case reports, reviews, abstracts, and studies without original data were excluded. For studies without raw data, we contacted the corresponding authors; if no response was received from the corresponding authors, the study was excluded.

### Study selection

A total of 1042 studies were initially obtained, and after exclusion of repeated citations using Endnote, 949 studies remained. Two reviewers independently screened all titles and abstracts to identify potentially eligible studies. The full text of these potentially eligible studies was then screened to determine the eligibility of the study for our meta-analysis. All differences were arbitrated by the corresponding author. Agreement between the two reviewers independently rating the articles was assessed using the kappa statistic at each step of selection. Finally, 33 studies were included in downstream analyses.

### Quality evaluation and data extraction

Study quality was estimated according to the Newcastle–Ottawa Scale (NOS) because the included studies primarily consisted of retrospective cohort studies. Each study was scored using the “star” rating system of NOS based on three aspects: the selection of the research population, the comparability of the study group, and the evaluation of the results. The scoring ranged from 0 to 9 stars. A study with a score ≥ 7 was considered to be high-quality research. Overall, the quality of the included studies based on the NOS was high (see Additional file 2).

Data were extracted in duplicate independently by two reviewers using an Excel form. Any differences were arbitrated by the corresponding author. The studies were characterized by the name of the authors, the year of publication, the location of the study, the period of the study, the total number of patients included, and the percentage of females. The baseline data included the average age of males and females, the location of where a patient suffered OHCA, whether there were witnesses to the OHCA, the proportion of cardiopulmonary resuscitation (bystander CPR), shockable rhythm, return of spontaneous circulation (ROSC), in-hospital intervention, and the existence of cardiogenic factors. The primary outcome was the survival from OHCA to discharge. The survival from OHCA to discharge is also generally considered the overall survival rate. In this meta-analysis, the overall survival rate was divided into two stages, namely the survival from OHCA to admission and the survival from admission to discharge. The secondary outcomes were the survival from OHCA to hospital admission, the survival from admission to discharge, and favorable neurological outcome. Favorable neurological outcome was evaluated by attending physicians and categorized according to the *Glasgow–Pittsburgh* cerebral *performance* categories (CPCs) 1 month after discharge. The outcomes were dichotomized as good (CPC 1, 2) and poor (CPC 3–5).

### Data analysis

The meta-analysis followed the guidelines outlined by the PRISMA 2009 checklist (see Additional file 3). The meta-analysis was performed using Stata software v. 15.0. Pooled odds ratios (ORs) and 95% confidence intervals (CIs) were estimated using a random-effects model. A Chi-squared test was used to evaluate the statistical heterogeneity between studies. Statistical heterogeneity across the studies was evaluated using the *I*^2^ statistic to quantify inconsistencies among studies that are not attributable to chance. Because of the characteristics of this retrospective cohort study, there is heterogeneity among the studies due to the influence of the baseline; therefore, the random-effect model was used. We analyzed the source of heterogeneity using a meta-regression analysis, where the dependent variable was the log odds ratio of survival and the independent variables were the above-mentioned baseline data.

Peters’ test was used to test potential publication biases. We also determined the quality of the original research using a sensitivity analysis. A *T* test was used for a baseline analysis.

## Results

**Fig. 1:**
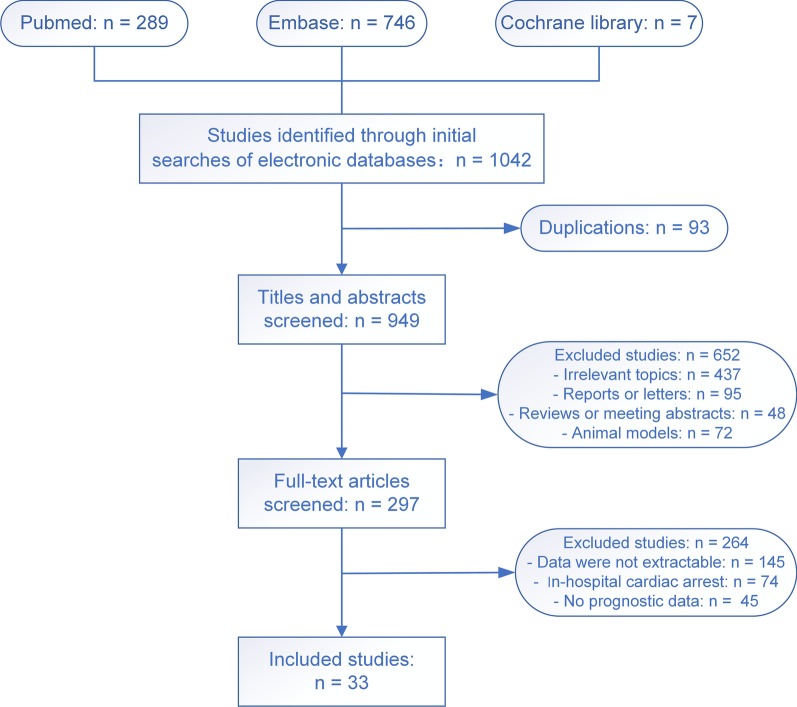
The process of study selection

Among the 1042 studies obtained in our initial search, 33 qualified articles (Fig. 1) were included in this meta-analysis. All 33 articles were full-text publications, from 34 countries and regions, with a total of 1,268,664 patients. All of the studies were retrospective cohort studies, and most of the data were obtained from local and national official databases. Agreement between the two reviewers was 94% for study selection and 91% for quality evaluation. No secondary analysis or additional publications were found related to the above-mentioned articles.

### Fundamental characteristics of research

The studies included were conducted between 1980 and 2018, and the study locations included four continents: 11 from America, 12 from Europe, nine from Asia, and two from Oceania. There were five studies with a small sample size (number < 1000) and 17 studies with a large sample size (number > 10,000). Among the 33 studies, 28 studies included non-traumatic OHCA and likely cardiac origin patients only, three studies lacked clear information on the aetiology of OHCA (see Additional file 4).

Table 1 shows the baseline level of this meta-analysis. Compared with males, females were older (69.7 years vs. 65.4 years, *p* < 0.05), suffered OHCA more often at home (73.44% vs 63.84%, *p* < 0.05), and were less likely to have witnesses to their OHCA (58.39% vs 62.70%, *p* < 0.05). In addition, the frequency of shockable rhythm in females was lower (25.74% vs 39.62%, *p* < 0.05). There were no differences in bystander resuscitation based on sex (38.82% vs 41.89%, *p* > 0.05) and ROSC (26.97% vs 26.10%, *p* > 0.05). However, females were less likely to receive in-hospital interventions such as percutaneous coronary intervention (PCI) (27.63% vs 34.78%, *p* < 0.05), coronary angiography (CAG) (37.84% vs 42.7%, *p* < 0.05), and target body temperature management (TTM) ( 25.05% vs 40.49%, *p* < 0.05) (Table 2).

**Table 1:** Studies included in the meta-analysis and baseline characteristics

References	Study location	Period	Total	Female (%)	Age (years)		OHCA at home (%)		Bystander-witnessed (%)		Bystander CPR (%)		Initial shockable rhythm (%)		Sustained ROSC (%)	
					F	M	F	M	F	M	F	M	F	M	F	M
Perman et al. [12]	California	2010–2011	6562	44.0	67.8	64.3										
Okabayashi et al. [22]	Japan	2013–2015	233,511	46.9												
Jeong et al. [7]	Korea	2006–2018	20,675	33.6	73.5	64.2	73.2	62.2	63	64.1	48.8	48	17.1	33.5	100	100
Goto et al. [13]	Japan	2013–2016	386,535	43.8	80.3	74.3			36.3	40.3	56.4	49.6	4.3	10.7		
Blom et al. [15]	Netherlands	2006–2012	5717	28.0	69.4	67.1	84.8	66.9	70.5	74.7	67.9	72.7	33.7	52.7	33.6	36.6
Matilde et al. [23]	Copenhagen	2007–2011	704	25.0	71	64	73.0	54.0	86.0	88.0	54.0	61.0	53.0	71.0	100	100
May et al. [24]	Detroit	2014–2016	2359	45.1												
Masterson et al. [25]	Sweden	2012–2014	15,303	33.4												
Hansen et al. [26]	North Carolina	2010–2014	8100	38.1	68	64	82.4	77	41.2	47.5	20.7	24.6	14.6	28.1		
Dicker et al. [11]	New Zealand	2013–2015	3862	31.0	68	65	75	63	49	55	59	64	28	43	29	31
Oh et al. [27]	Korean	2007–2012	930	30.1	56.9	56.7			62.1	68.9	27.5	31.4	20	33.1	100	100
Hagihara et al. [28]	Japan	2005–2012	24,216	13.2	52.3	53.1			73.4	76.3			100	100	34	27.9
Bougouin et al. [29]	France	2000–2013	1817	28.6	62.8	59.1	57.0	37.0	84.0	88.0	45.0	48.0	42.0	61.0	100	100
Ng et al. [30]	Pan-Asian	2009–2012	40,159	40.0	82	72	32.0	32.5	38.0	44.6	41.2	36.6	6.7	17.7	6.1	10
Morrison et al. [31]	Canada and Amercia	2000–2006	14,690	36.4	73	66	92	79.4	34.3	41.8	29.6	31.0	16.6	30.0	28.1	28.2
Bosson et al. [32]	Los Angeles	2011–2014	5174	40.5	71	66			81.0	83.0	39.0	41.0	22.0	35.0		
Karlsson et al. [33]	Europe and the USA	2006–2012	1667	28.0	62	63			80.0	83.0	58.0	64.0	52.0	69.0	100	100
Wissenberg et al. [34]	Danish	2001–2010	19,372	32.6	75	70	81.3	70.6	49.1	53.4	25.9	32.9	17.2	32.6		
Safda et al. [35]	Ontario	1994–2002	11,479	32.5	74	69			42.8	48.7	12.1	16.9	24.4	41.6	11.8	11.1
Johnson et al. [36]	America	2005–2009	19,398	39.5	69	63	92.8	83.5	44.8	49.9	33.3	33.7	16.5	28.5	34.9	32.3
Bray et al. [37]	Australian	2003–2010	10,453	30.0	74	69	76.0	68.0	57.0	63.0	48.0	52.0	26.0	45.0		
Teodorescu et al. [38]	America	2002–2007	1296	33.0	68	63	69.0	61.0	66.0	68.0	24.0	28.0	38.0	55.0	41.3	33.0
Ahn et al. [39]	Korea	2007–2008	13,922	37.0	72.4	63.7	82.8	71.8	45	46.9			3.4	5.4		
Akahane et al. [40]	Japan	2005–2007	318,123	37.8	72.7	67.9			36.9	42.1	38.3	32.8	4.7	10.1		
Adielsson et al. [41]	Sweden	1990–2009	7187	19.0	71	69	60.0	53.0	51.0	56.0	100	100	100	100		
Kitamura et al. [16]	Japan	1998–2007	26,940	41.5	78.7	72.0	75.0	70.0	34.0	41.0	28.0	34.0	6.0	13.0		
Arrich et al. [42]	Austria	1991–2004	801	25.6	62	60			100	100	29.0	37.0	64.0	78.0		
Mahapatra et al. [43]	Olmstead	1990–2000	200	18.5	65	64			86.0	82.0	46.0	57.0	100	100		
Cline et al. [44]	America	1997–1999	388	35.6	69.3	64	75.0	75.0	44.0	58.0	51.0	52.0	33.0	54.0	23.9	24.8
Herlitz et al. [45]	Swedish	1990–2000	23,797	27.9	69	67	72.0	63.0	62.0	64.0	32.0	37.0	24.0	36.0		
Kim et al. [46]	America	1990–1998	10,879	35.0	71	66	75.0	70.0	48.0	54.0	45.0	47.0	25.0	43.0		
Pell et al. [47]	Scotland	1988–1997	22,161	30.0	69	65	67.0	55.0	58.0	63.0			49.0	59.0		
Perers et al. [48]	Go¨ teborg	1980–1996	4401	28.0	73	69			70.0	73.0	11.0	15.0	28.0	44.0

**Table 2:** The sex difference in post-resuscitation care

References	PCI (%)		TTM (%)		CAG (%)	
	F	M	F	M	F	M
Perman et al. [12]	3.84	9.47			9.30	18.15
Jeong et al. [7]	3.30	12.10	7.50	11.30		
Blom et al. [15]	8.75	14.10	24.90	26.20	16.44	25.30
Matilde et al. [23]	17	30	47	61	58	88
Hansen et al. [26]			58.50	62.40	23.70	41.40
Dicker et al. [11]	68	64				
Oh et al. [27]			100	100	7.90	15.50
Bougouin et al. [29]	17.12	31.60	70.00	72.00	49.00	70.00
Bosson et al. [32]	47.00	54.00	33.00	40.00	11.00	25.00
Karlsson et al. [33]			100	100		
Bray et al. [37]	56.00	63.00				
Arrich et al. [42]			24.00	26.00		

*F* females, *M* males, *PCI* percutaneous coronary intervention, *TTM* target body temperature management, *CAG* coronary angiography

Among the 33 studies, 15 studies had data on admission survival rate between the sexes, nine studies had data on survival from admission to discharge, 27 studies had data on survival to discharge, and 15 studies included data on nervous system prognosis between the sexes.

### Meta-analysis

#### Sex differences in survival after OHCA

**Fig. 2:**
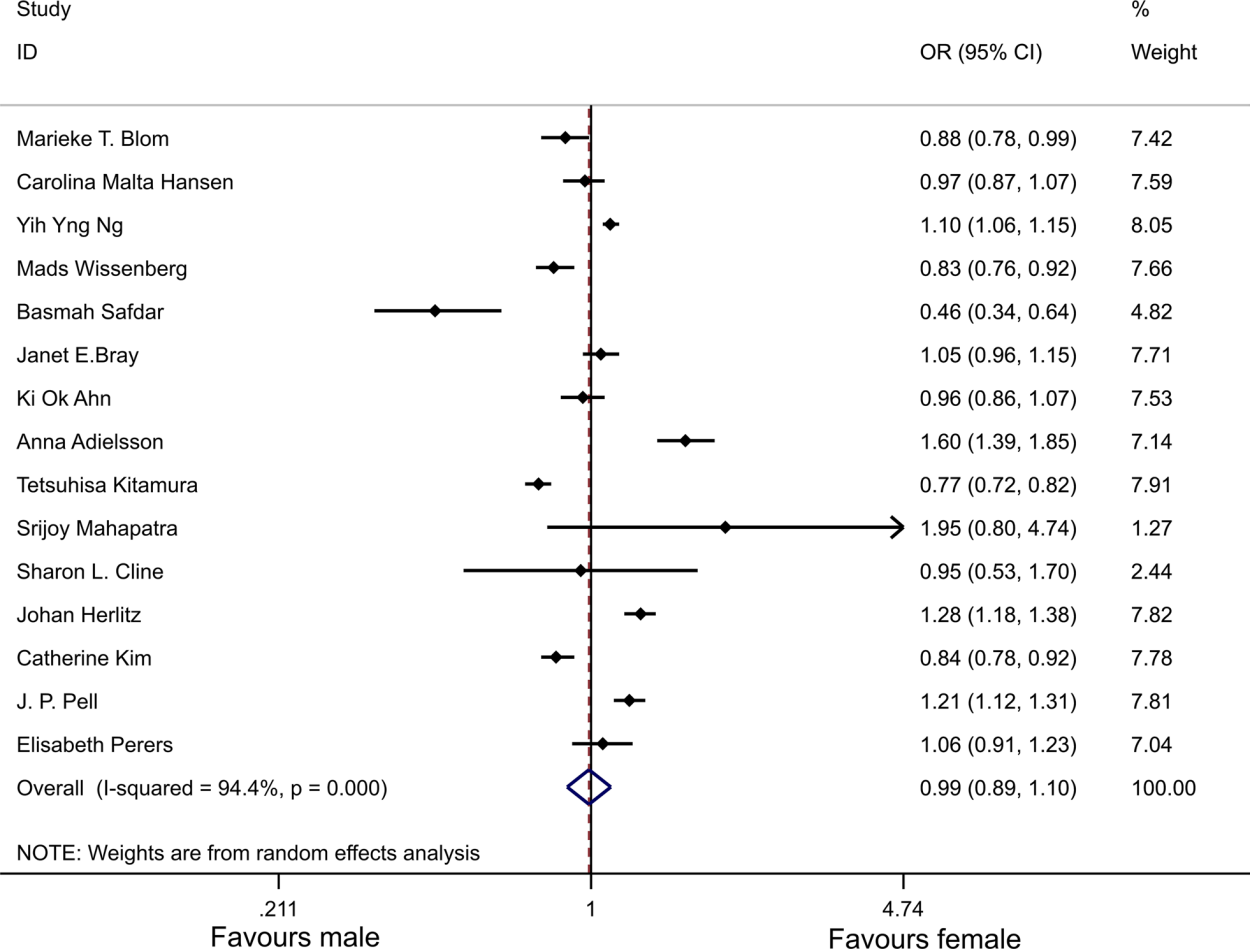
Forest plot and meta-analysis of the sex differences in survival from OHCA to admission

Fifteen of the 33 studies were used to analyze the differences in survival from OHCA to admission, and we found no significant difference in survival from OHCA to admission between males and females (OR 0.99, 95% CI 0.89–1.1) (Fig. 2). Heterogeneity analysis showed there was heterogeneity among 15 studies (*χ*^2^ = 247.82, df = 14, *p* < 0.001; *I*^2^ = 94.4%).

**Fig. 3:**
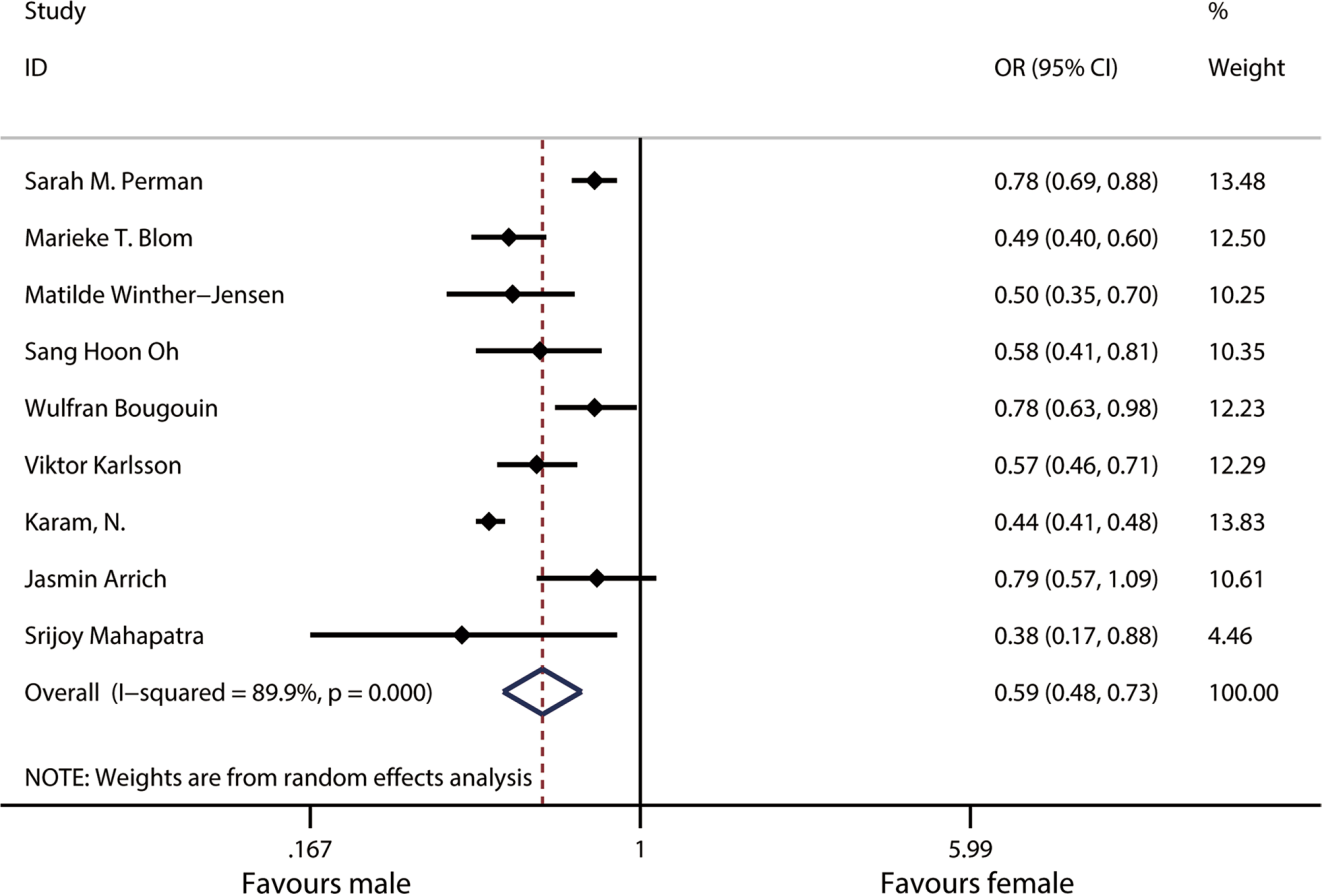
Forest plot and meta-analysis of the sex differences in survival from admission to discharge

A total of nine studies were analyzed to determine the difference in sex related to the survival from admission to discharge. The results showed that the survival from admission to discharge was significantly lower for females than for males (OR 0.59, 95% CI 0.48–0.73) (Fig. 3). Heterogeneity analysis showed there was heterogeneity among nine studies (*χ*^2^ = 79.14, df = 8, *p* < 0.001; *I*^2^ = 89.9%).

**Fig. 4:**
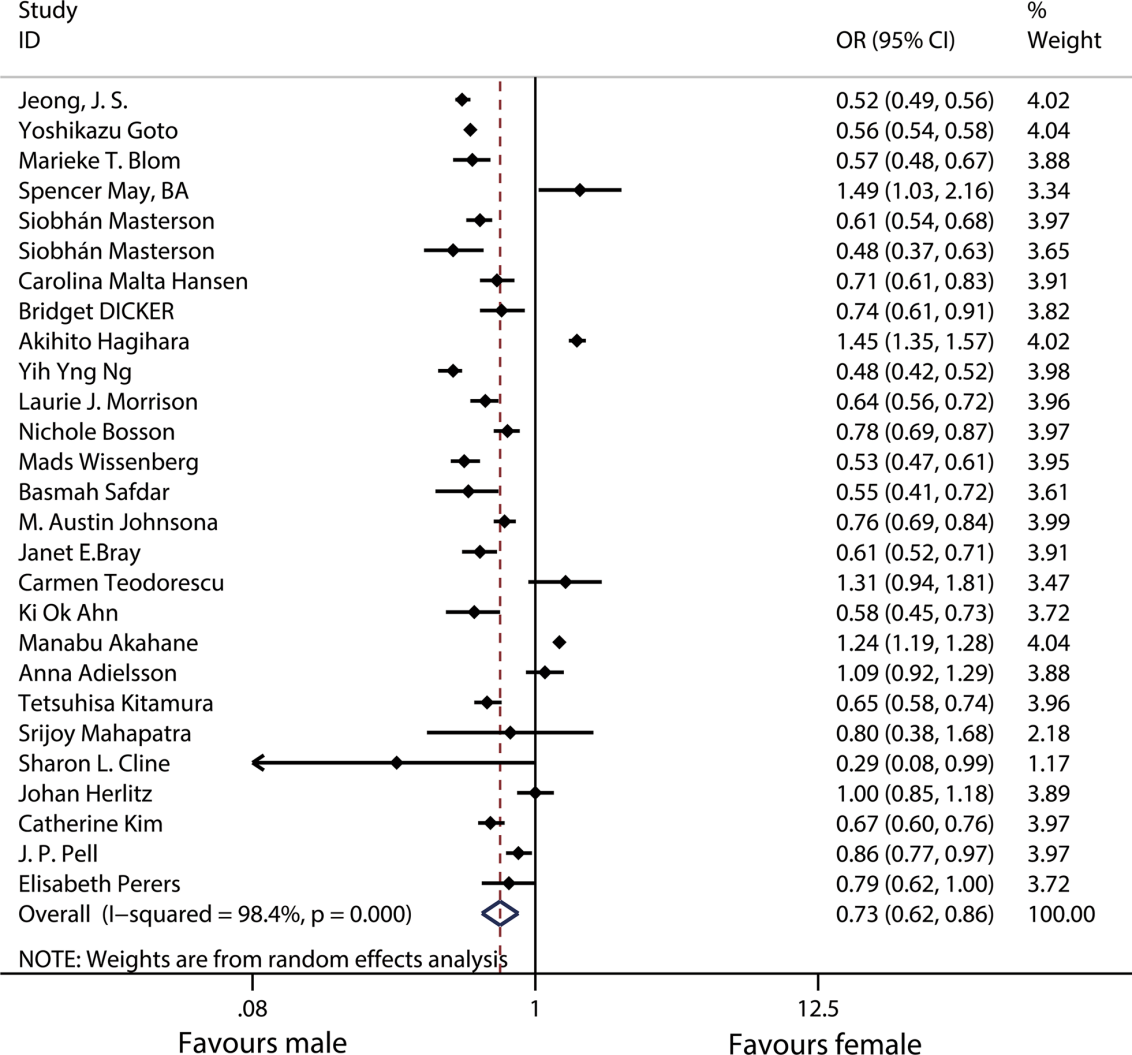
Forest plot and meta-analysis of the sex differences in survival from OHCA to discharge

A total of 27 studies were analyzed to determine the differences in survival from OHCA to discharge. The results showed that the survival to discharge was significantly lower for females than for males (OR 0.73, 95% CI 0.62–0.86) (Fig. 4). Heterogeneity analysis showed there was heterogeneity among 27 studies (*χ*^2^ = 1607.1, df = 26, *p* < 0.001; *I*^2^ = 98.4%).

#### Sex differences in neurological prognosis

**Fig. 5:**
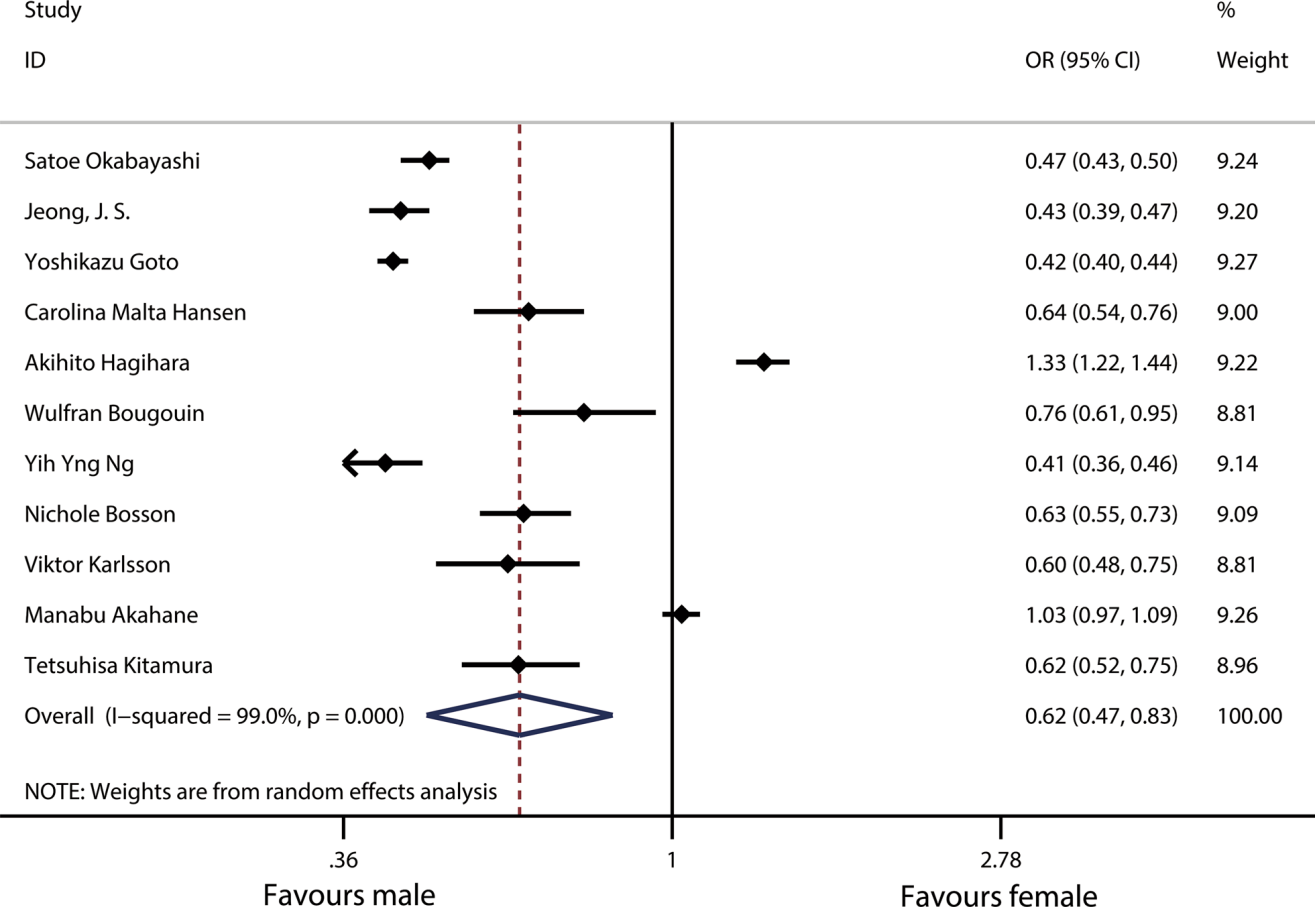
Forest plot and meta-analysis of the sex differences in neurological prognosis

Based on the comprehensive analysis of the neurological prognosis of 11 studies related to OHCA, a CPC score of 1–2 was regarded as a good prognosis. The results showed that the neurological prognosis of females after OHCA was not as optimistic as that of males; specifically, females had a lower probability of good neurological prognosis than males (OR 0.62, 95% CI 0.47–0.83) (Fig. 5). Heterogeneity analysis showed there was heterogeneity among 11 studies (*χ*^2^ = 1029.79, df = 10, *p* < 0.001; *I*^2^ = 99%).

### Sensitivity analysis and publication bias

Sensitivity analyses of the above four outcomes did not suggest that missing studies affected any conclusions appreciably (see Additional file 5).

To avoid Type I error, we assessed publication bias by Peters’ test. The *p* value of Peter’s test was 0.867, 0.831, 0.959, and 0.967. There was no evidence of publication bias.

### Meta-regression

Considering the significant heterogeneity of above four outcomes, we conducted a meta-regression analysis to explore the source of heterogeneity. Table 3 shows the result of meta-regression. There was no significant association between rates of OHCA at home, of bystander-witnessed, of bystander CPR, and survival (*p* > 0.05). However, there was a significant correlation between the rate of initial shockable rhythm and prognosis (*p* < 0.05). Additionally, there is also a significant correlation between the rate of ROSC and survival from OHCA to discharge.

**Table 3:** The *p* value of meta-regression

	OHCA at home	Bystander-witnessed	Bystander CPR	Initial shockable rhythm	Sustained ROSC
A	0.125	0.156	0.34	0.039	0.35
B	0.496	0.116	0.906	0.001	0.012
C	0.283	0.672	0.72	0.026	0.117

A = meta-analysis of the sex differences in survival from OHCA to admission; B = meta-analysis of the sex differences in survival from OHCA to discharge; C = meta-analysis of the sex differences in neurological prognosis

## Discussion

In our baseline statistical analysis of OHCA patients, we found that females are older, which may be associated with coronary artery disease occurring 10–15 years later in females than in males; it may also be related to the longer life expectancy of females than males [5]. At the same time, females are more likely to have cardiac arrest at home than in public, which may be because females spend more time at home than males. As a result, females are less likely to be witnessed by bystanders during OHCA. Previous studies have shown that even when both males and females experience cardiac arrest with bystander witnesses, females are less likely to receive CPR because of conservative attitudes and privacy concerns [15]. However, our findings were not in agreement; we found no statistically significant difference in the proportion of females and males who received bystander CPR. This might be related to regional and temporal differences of the studies included in this meta-analysis. In two clinical studies in Japan, for example, data from 1998 to 2007 showed that the proportion of females and males receiving bystander CPR after OHCA was 28% and 34% [16], while data from 2013 to 2016 showed that the proportion of bystander CPR increased significantly, and the proportion of females receiving bystander CPR was even higher than males (56.4% vs 49.6%). We speculate that with the development of society, the comprehensive popularization of CPR-related knowledge, and the change of stereotypes, an increasing number of bystanders can perform CPR, which not only increase the proportion of bystander CPR, but also make up for the corresponding sex differences.

Previous meta-analyses have shown that factors, such as age, bystander witnesses, bystander CPR, and shockable rhythm, are all associated with the outcome of OHCA [17]. Our baseline statistics also showed some differences between females and males, but our analysis showed that there was no significant difference in survival between females and males after OHCA until admission. Even though the treatment measures were different from OHCA to admission, they had little effect on the survival at admission. Therefore, we speculate that some pre-hospital baseline factors (age, bystander witness, bystander CPR, and shockable rhythm) do not have an obvious immediate impact on the differences in survival from OHCA to admission between the sexes. Moreover, there may be corresponding long-term effects, such as affecting survival after discharge and nervous system prognosis of patients.

Our meta-analysis showed that after cardiac arrest, the survival of females from admission to discharge was significantly lower than that of males even considering no significant sex difference in survival at admission. The overall discharge survival rate for females remains much lower than that for males, and the neurological prognosis for females is also poor. Our fundamental analysis showed that females generally received less post-admission interventions, such as PCI, CAG, and TTM, than males. Previous studies have shown that women are significantly less likely to receive PCI after OHCA than men, although the prognosis after PCI is not significantly related to sex [7]. Therefore, the survival of females after OHCA is lower than males, which may be related to the differences in medical services received after admission.

In contrast, some studies have suggested that female hormones are the dominant factor in OHCA because estrogen can resist apoptosis and inflammation. Estrogen has a protective effect on heart and nerves, but this has not been widely confirmed in clinical practice [18]. We believe that although females have estrogen as a favorable prognostic factor, there are also corresponding unfavorable factors, such as age, pathophysiology, acceptance of medical services, and willingness to express opinion. First, females are older at onset and more likely to have various complications, such as chronic obstructive pulmonary disease (COPD), tumor, and psychiatric history. Secondly, female pathophysiological differences make them more prone to complications such as bleeding [19] and acute renal failure [20]. Females have a lower acceptance of corresponding medical services, drug treatment, and invasive procedures. Additionally, females generally receive less corresponding nursing measures. For example, during OHCA, females were less likely than males to receive intravenous access and intraosseous access [8]. This may be related to the fact that nurses perceived intravenous access as more difficult in females than in males, although there was no difference in success rate. Finally, females are more willing to express their intentions and participate in the discussion about their death [21]. To a certain extent, these factors will affect the choice of resuscitation measures.

Combined with factors, such as education level, religious belief, and economic level, females are more likely to issue the instruction of "Do not attempt resuscitation" at the early stage of OHCA, thus adopting more conservative treatment [12]. Overall, this leads to a poor prognosis of OHCA in females.

Our meta-analysis also had some limitations. We have only searched three major databases, and there may be studies that are not included. Specifically, it was limited by the type of study; therefore, we could only analyze the correlation of various factors, but could not directly analyze the causal relationship. Moreover, the types of studies included were all observational studies, so it was difficult to control the corresponding confounding factors. For example, the time and location of studies were uncontrollable confounding factors. As mentioned above, the studies included were conducted between 1980 and 2018. However, post-resuscitation care guidelines have changed over the years these studies were conducted, especially in terms of TTM. On the other hand, the included studies were distributed in different emergency medical systems, and these emergency medical systems have different medical levels and different termination of resuscitation rule, which may affect the survival rate of OHCA. Based on the above factors, the heterogeneity of our meta-analysis is relatively high. However, we conducted meta-regression and sensitivity analysis to find the main source of heterogeneity and to ensure the reliability of the results. Finally, post-resuscitation care research is all affected by immortal time bias. Immortal time bias likely results in apparent lower mortality rates and thus overestimates of the potential benefits on survival.

## Conclusions

Our results showed that after OHCA, the overall discharge survival rate of females was much lower than males and the prognosis of the nervous system was poor. We speculate that this may be related to different medical services and nursing measures; however, these findings should be interpreted with caution due to the presence of several confounding factors. Many of these adverse conditions for females can be changed with education, so our results also provide new insights and directions for how to narrow the difference in prognosis between males and females after OHCA.

## Supplementary information

**Additional file 1**. Search strategy.

**Additional file 2**. Quality evaluation.

**Additional file 3**. PRISMA checklist.

**Additional file 4**. The aetiology of OHCA in the population included in the study.

**Additional file 5**. Sensitivity analyses.

## Data Availability

All data generated or analyzed during this study are included in this published article.

## Abbreviations

OHCA: Out-of-hospital cardiac arrest
EMS: Emergency medical service
ROSC: Return of spontaneous circulation
PCI: Percutaneous coronary intervention
CAG: Coronary angiography
TTM: Target body temperature management
