# Supplementary material for: Sex differences in survival after out-of-hospital cardiac arrest: a meta-analysis
Source: Crit Care. 2020 Oct 19;24:613. doi: 10.1186/s13054-020-03331-5 (PMC7570116; doi:10.1186/s13054-020-03331-5)
Supplement: Supplementary file 1 — Additional file 1. Search strategy. [file 13054_2020_3331_MOESM1_ESM.docx]

Search strategy

A combination of keywords related to:

(1) Type of exposure: “women” or “female” or “men” or “male” or “sex” or “gender” ; (2) Cardiac arrest: “sudden death” or “cardiac arrest” or “out-of-hospital SCA” or “sudden cardiac death” or “sudden cardiac arrest” or “Out-of-hospital cardiac arrest” or “cardioplegic arrest” or “sudden heart arrest” or “out of hospital cardiac arrest”.

(3) Outcome: “survival” or “prognosis” or “outcome” or “viability” or “prognostic” or “ending” or “mortality” or “outcomes” or “endings”.

Search ((((((((((((Out-of-hospital cardiac arrest[MeSH Terms]) OR Heart Arrest[MeSH Terms]) OR Cardiac arrest[Title]) OR Sudden death[Title]) OR Out-of-hospital SCA[Title]) OR Sudden cardiac death[Title]) OR Sudden cardiac arrest[Title]) OR sudden heart arrest[Title]) OR cardioplegic arrest[Title]) OR out of hospital cardiac arrest[Title])) AND ((((((sex[Title]) OR gender[Title]) OR women[Title]) OR men[Title]) OR female[Title]) OR male[Title])) AND (((((((((((((Prognosis[MeSH Terms]) OR Survival Rate[MeSH Terms]) OR Mortality[MeSH Terms]) OR (Outcome and Process Assessment (Health Care)[MeSH Terms])) OR survival[Title/Abstract]) OR Prognosis[Title/Abstract]) OR Outcome[Title/Abstract]) OR Viability[Title/Abstract]) OR Prognostic[Title/Abstract]) OR Ending[Title/Abstract]) OR Outcomes[Title/Abstract]) OR Endings[Title/Abstract]) OR Mortality[Title/Abstract])
