# Supplementary material for: Sex differences in survival after out-of-hospital cardiac arrest: a meta-analysis
Source: Crit Care. 2020 Oct 19;24:613. doi: 10.1186/s13054-020-03331-5 (PMC7570116; doi:10.1186/s13054-020-03331-5)
Supplement: Supplementary file 2 — Additional file 2. Quality evaluation. [file 13054_2020_3331_MOESM2_ESM.docx]

| NEWCASTLE-OTTAWA SCALE COHORT STUDIES (http://www.ohri.ca/programs/clinical_epidemiology/oxford.asp) | | | | | | | | | | |
| --- | --- | --- | --- | --- | --- | --- | --- | --- | --- | --- |
| Author | Year | ***Selection—***  ***Representativeness of exposed cohort*** | ***Selection—***  ***Representativeness of the unexposed cohort*** | ***Selection—***  ***Ascertainment of exposure*** | ***Selection—Demonstration,outcome of the study, was not present at the beginning of the study*** | ***Comparability of cohorts*** | ***Outcome– Assessment of outcome*** | ***Outcome– Follow-up long enough*** | ***Outcome–***  ***Adequacy of follow-up cohorts*** | ***Quality rating*** |
| ***Retrospective cohort*** | | | | | | | | | | |
| Perman | 2019 | * | * | * | * | - | ** | * | * | Good |
| Okabayashi | 2019 | * | * | * | * | * | * | - | * | Good |
| Jeong | 2019 | * | * | * | * | - | * | * | * | Good |
| Goto | 2019 | * | * | * | * | * | * | * | * | Good |
| Blom | 2019 | * | * | * | * | * | * | * | * | Good |
| Jensen | 2019 | * | * | * | * | - | * | * | * | Good |
| May | 2019 | * | * | * | * | * | * | * | * | Good |
| Masterson | 2018 | * | * | * | * | * | * | * | * | Good |
| Hansen | 2018 | * | * | * | * | * | * | * | * | Good |
| Dicker | 2018 | * | * | * | * | * | * | * | * | Good |
| Oh | 2017 | * | * | * | * | * | * | * | * | Good |
| Hagihara | 2017 | * | * | * | * | - | * | * | * | Good |
| Bougouin | 2017 | * | * | * | * | * | * | * | * | Good |
| Ng | 2017 | * | * | * | * | * | * | * | * | Good |
| Morrison | 2016 | * | * | * | * | * | * | * | - | Good |
| Bosson | 2016 | * | * | * | * | * | * | * | - | Good |
| Karlsson | 2015 | * | * | * | * | - | * | * | - | Good |
| Wissenberg | 2014 | * | * | * | * | - | * | * | - | Good |
| Safdar | 2014 | * | * | * | * | - | * | * | * | Good |
| Johnsona | 2013 | * | * | * | * | * | * | * | - | Good |
| Bray | 2013 | * | * | * | * | * | * | * | * | Good |
| Teodorescu | 2012 | * | * | * | * | * | * | * | * | Good |
| Ahn | 2012 | * | * | * | * | * | * | * | * | Good |
| Akahane | 2011 | * | * | * | * | * | * | * | * | Good |
| Adielsson | 2011 | * | * | * | * | * | * | * | - | Good |
| Kitamura | 2010 | * | * | * | * | * | * | * | * | Good |
| Arrich | 2006 | * | * | * | * | - | * | * | * | Good |
| Mahapatra | 2005 | * | * | * | * | - | * | * | * | Good |
| Cline | 2005 | * | * | * | * | * | * | * | * | Good |
| Herlitz | 2004 | * | * | * | * | - | * | * | * | Good |
| Kim | 2001 | * | * | * | * | * | * | * | * | Good |
| Pell | 2000 | * | * | * | * | - | * | * | * | Good |
| Perers | 1999 | * | * | * | * | * | * | * | * | Good |
