# Supplementary material for: Sex differences in survival after out-of-hospital cardiac arrest: a meta-analysis
Source: Crit Care. 2020 Oct 19;24:613. doi: 10.1186/s13054-020-03331-5 (PMC7570116; doi:10.1186/s13054-020-03331-5)
Supplement: Supplementary file 4 — Additional file 4. The aetiology of OHCA in the population included in the study. [file 13054_2020_3331_MOESM4_ESM.docx]

| Year | Author | Study location | The aetiology of OHCA |
| --- | --- | --- | --- |
| 2019 | Sarah M. Perman | California | all |
| 2019 | Satoe Okabayashi | Japan | non-traumatic OHCA |
| 2019 | Jeong, J. S. | Korea | cardiac origin |
| 2019 | Yoshikazu Goto | Japan | non-traumatic OHCA |
| 2019 | Marieke T. Blom | Netherlands | cardiac origin |
| 2019 | Matilde Winther-Jensen | Copenhagen | cardiac origin |
| 2018 | Spencer May, BA | Detroit | non-traumatic OHCA |
| 2018 | Siobhán Masterson | Ireland | non-traumatic OHCA |
| 2018 | Carolina Malta Hansen | North Carolina | cardiac origin |
| 2018 | Bridget DICKER | New Zealand | unknow |
| 2017 | Sang Hoon Oh | Korean | non-traumatic OHCA |
| 2017 | Akihito Hagihara | Japan | non-traumatic OHCA |
| 2017 | Wulfran Bougouin | France | non-traumatic OHCA |
| 2017 | Yih Yng Ng | Pan-Asian | cardiac origin |
| 2016 | Laurie J. Morrison | Canada and Amercia | non-traumatic OHCA |
| 2016 | Nichole Bosson | Los Angeles | unknow |
| 2015 | Viktor Karlsson | Europe and the USA | non-traumatic OHCA |
| 2014 | Mads Wissenberg | Danish | cardiac origin |
| 2014 | Basmah Safdar | Ontario | cardiac origin |
| 2013 | M. Austin Johnsona | America | cardiac origin |
| 2013 | Janet E.Bray | Australian | cardiac origin |
| 2012 | Carmen Teodorescu | USA | cardiac origin |
| 2012 | Ki Ok Ahn | Korea | cardiac origin |
| 2011 | Manabu Akahane | Japan | all |
| 2011 | Anna Adielsson | Sweden | cardiac origin |
| 2010 | Tetsuhisa Kitamura | Japan | cardiac origin |
| 2006 | Jasmin Arrich | Austria | cardiac origin |
| 2005 | Srijoy Mahapatra | Olmstead | cardiac origin |
| 2005 | Sharon L. Cline | USA | non-traumatic OHCA |
| 2004 | Johan Herlitz | Swedish | cardiac origin |
| 2001 | Catherine Kim | USA | non-traumatic OHCA |
| 2000 | J. P. Pell | Scotland | unknow |
| 1999 | Elisabeth Perers | Go¨ teborg | cardiac origin |
