# Supplementary material for: Sex differences in survival after out-of-hospital cardiac arrest: a meta-analysis
Source: Crit Care. 2020 Oct 19;24:613. doi: 10.1186/s13054-020-03331-5 (PMC7570116; doi:10.1186/s13054-020-03331-5)
Supplement: Supplementary file 5 — Additional file 5. Sensitivity analyses. [file 13054_2020_3331_MOESM5_ESM.pptx]

## Slide 1
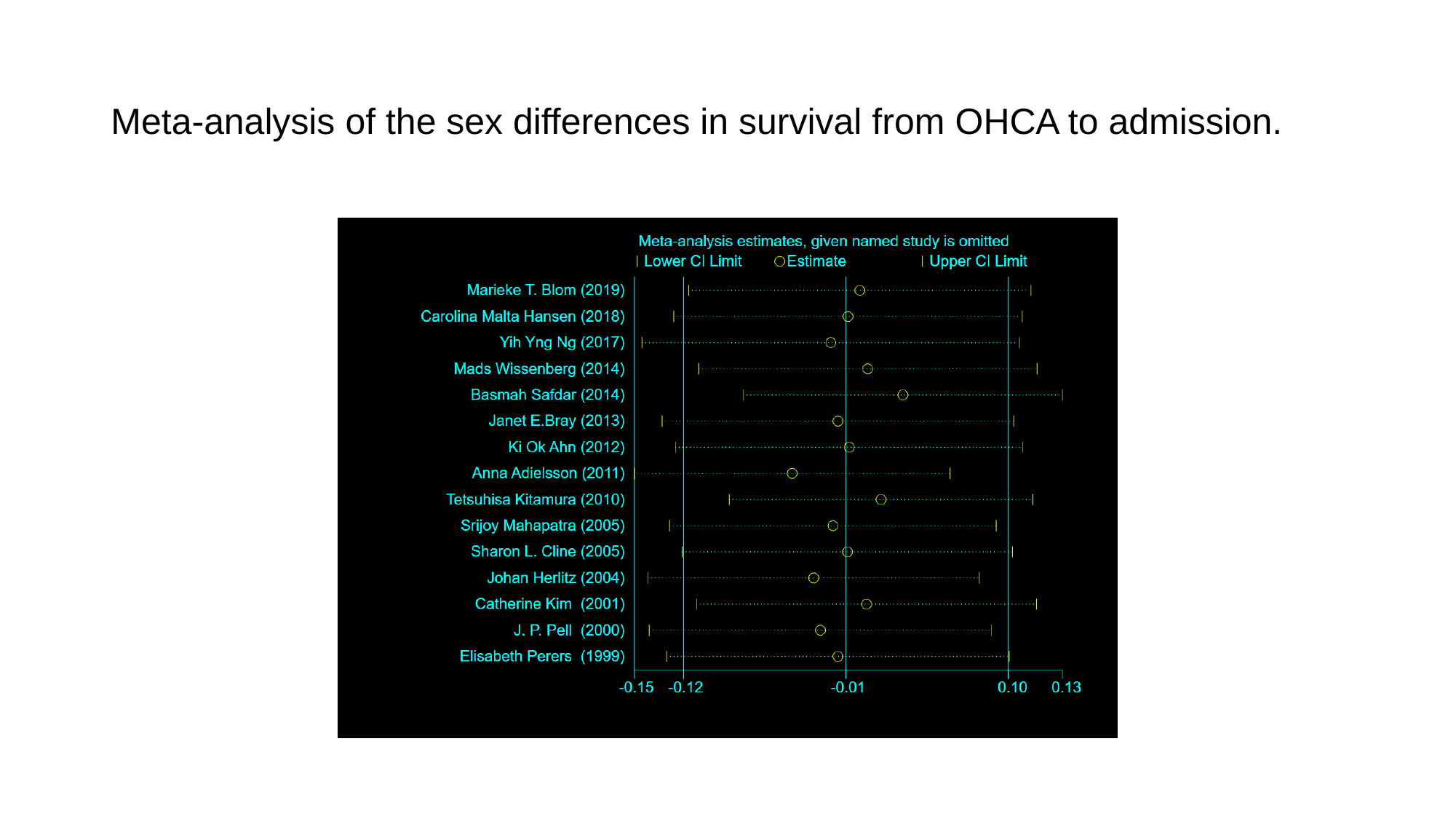

# Meta-analysis of the sex differences in survival from OHCA to admission.

## Slide 2
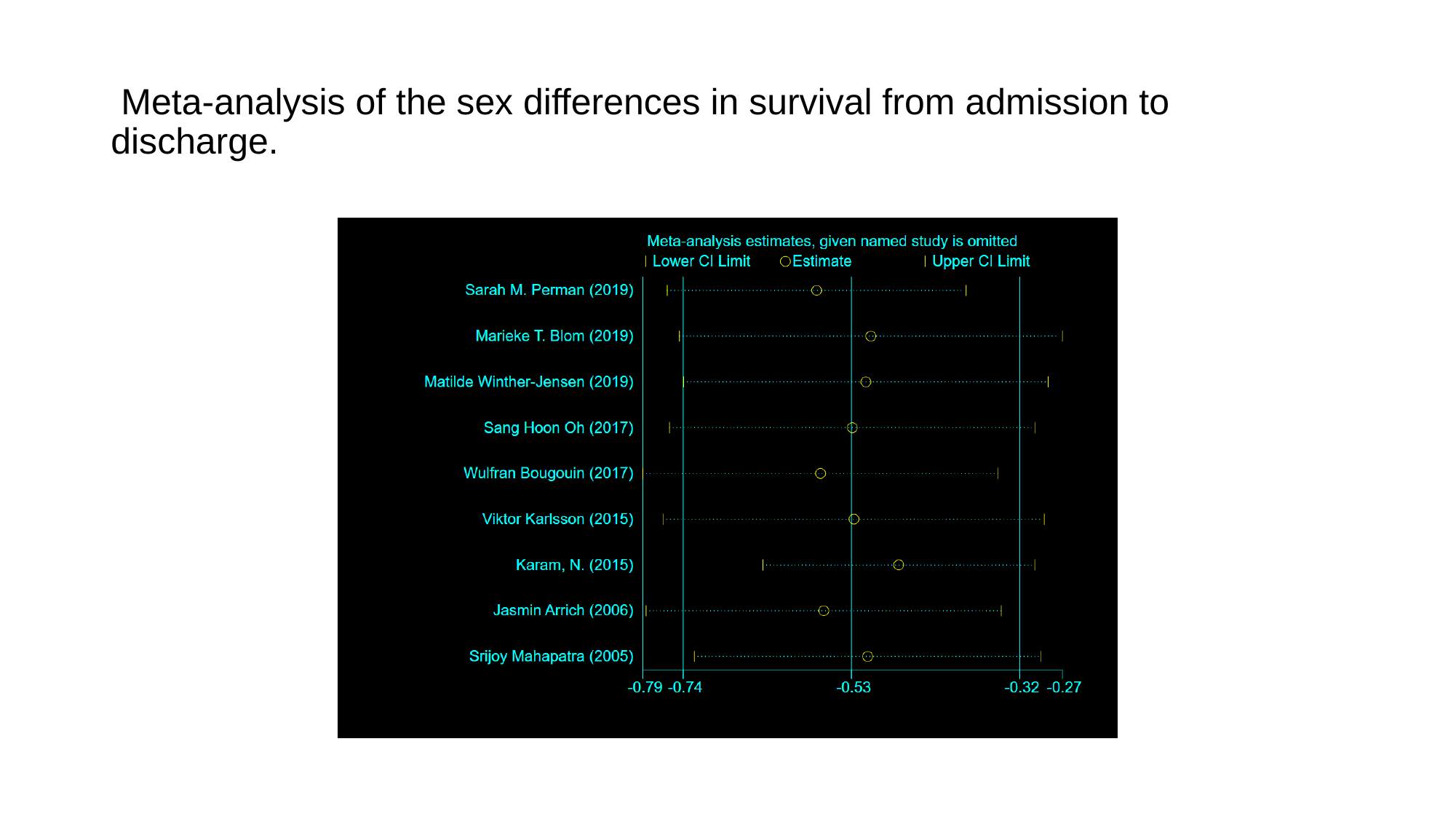

# Meta-analysis of the sex differences in survival from admission to discharge.

## Slide 3
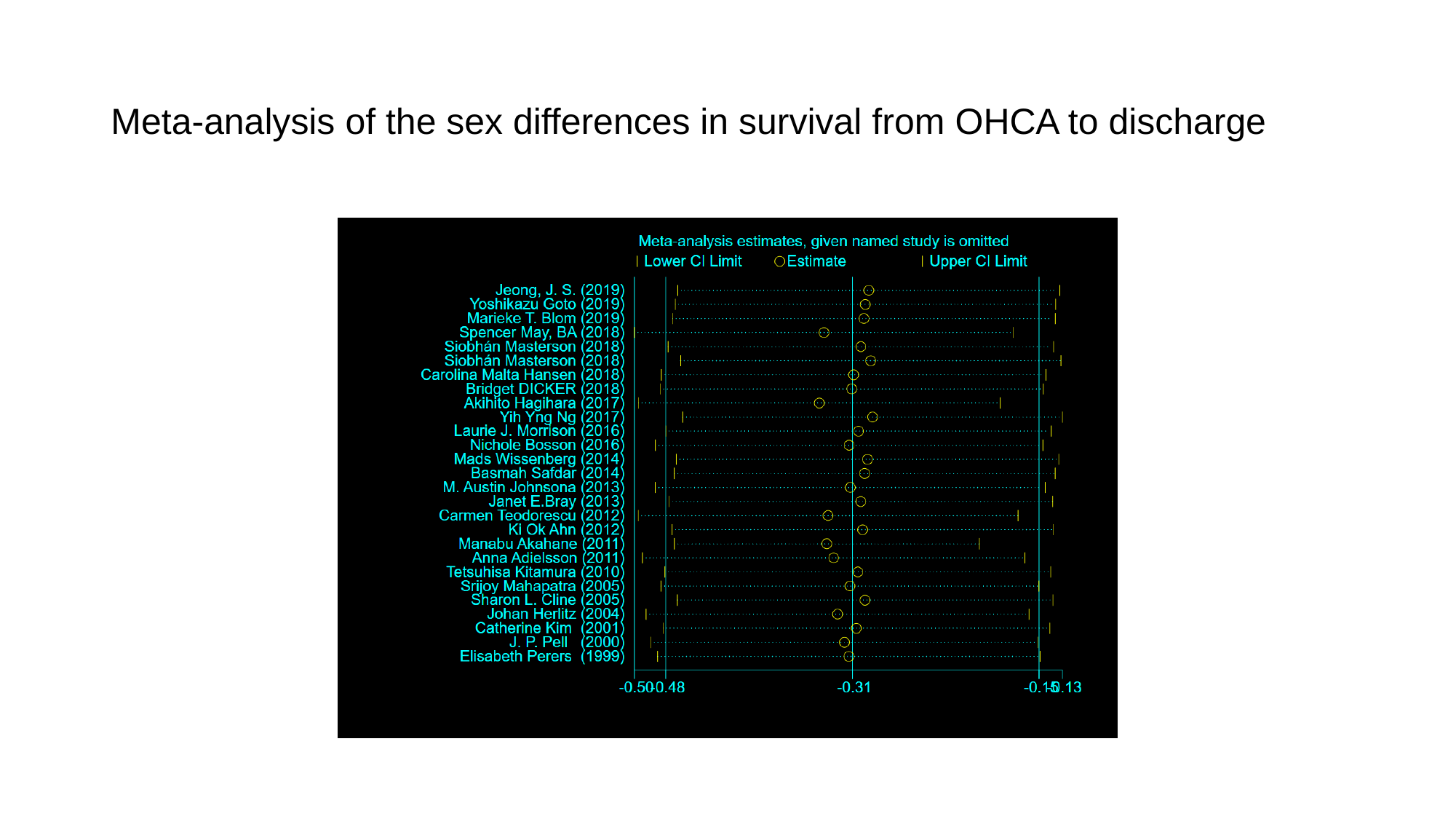

# Meta-analysis of the sex differences in survival from OHCA to discharge

## Slide 4
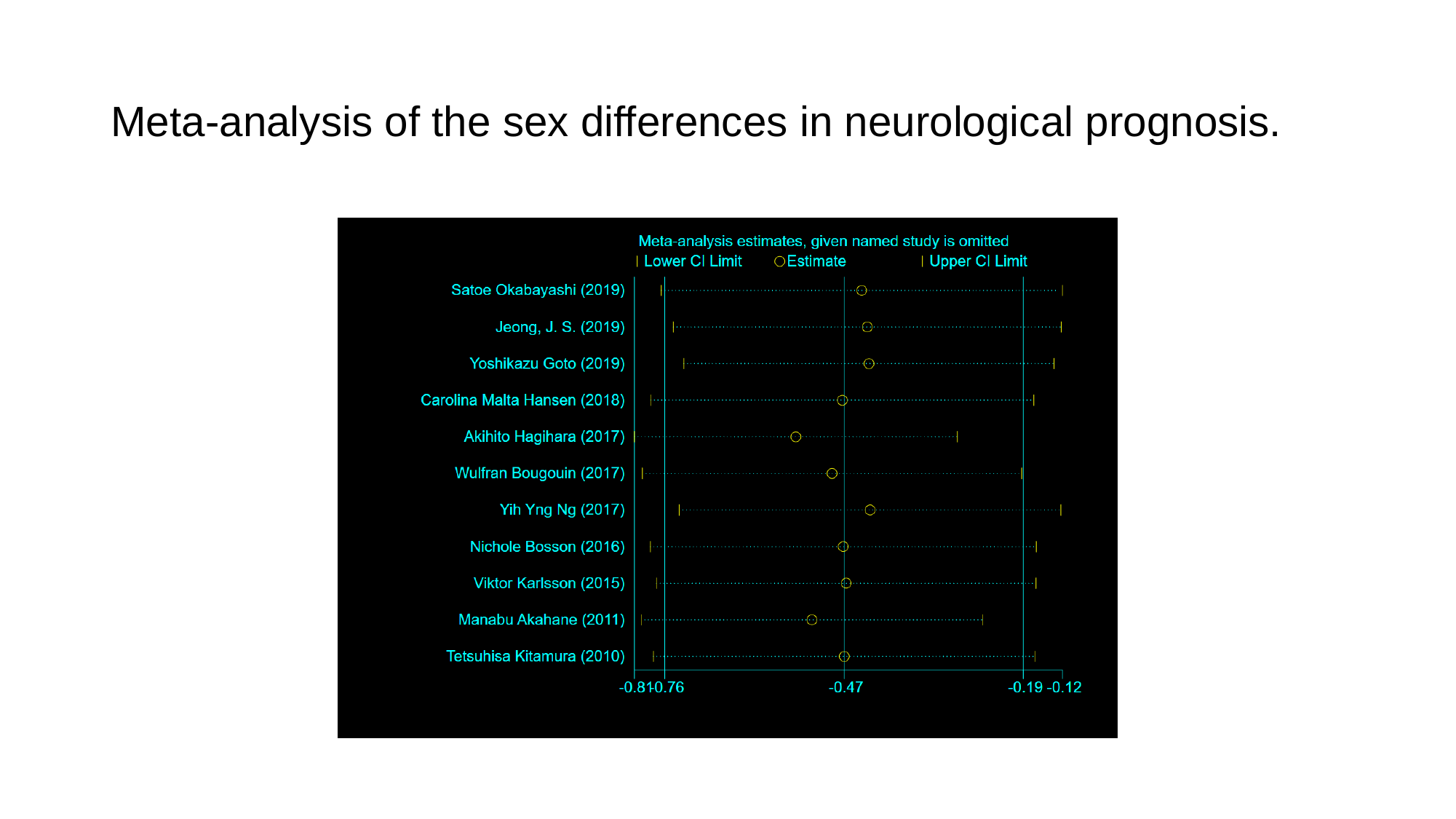

# Meta-analysis of the sex differences in neurological prognosis.
